# The impact of root systems and their exudates in different tree species on soil properties and microorganisms in a temperate forest ecosystem

**DOI:** 10.1186/s12870-024-04724-2

**Published:** 2024-01-12

**Authors:** Karolina Staszel-Szlachta, Jarosław Lasota, Andrzej Szlachta, Ewa Błońska

**Affiliations:** 1 Department of Ecology and Silviculture, Faculty of Forestry, University of Agriculture in Krakow, 29 Listopada 46 Str, 31-425 Krakow, Poland; 2 Swierklaniec Forest District, Ul. Oswiecimska 19, 42-622 Swierklaniec, Poland

**Keywords:** Enzyme activity, Fungal and bacterial diversity, Next-generation sequencing (NGS), Root characteristics

## Abstract

**Background:**

The species composition of tree stands plays an important role in shaping the properties of forest soils. The aim of our research was to determine the influence on soil properties of the root systems of six species of trees which form forest stands in the temperate climatic zone. The research covered areas including six tree species – Scots pine (*Pinus sylvestris L.*), European larch (*Larix deciduas Mill*.), English oak (*Quercus robur L.),* English ash (*Fraxinus excelsior L.),* European beech (*Fagus sylvatica L*.) and European hornbeam (*Carpinus betulus L.).* In our study, we determined the characteristics of the roots and the amount of carbon excreted alongside their exudates. Enzymatic activity, and the composition and diversity of the fungi and bacteria, were also determined in addition to the basic physicochemical properties of the soil samples.

**Results:**

A strong relationship between the root characteristics and soil properties, including the pH, basic cation content and phosphorus content, was confirmed. In addition, the enzymatic activity of phosphatase, β-glucosidase, N-acetyl-β-D-glucosaminidase and β-D-cellobiosidase were positively correlated with the root characteristics. The study on soil bacteria across different tree species revealed *Proteobacteria* and *Actinobacteriota* to be the most abundant phylum. Fungal analysis showed *Basidiomycota* and *Ascomycota* as the dominant phyla. *Ascomycota* dominated in hornbeam and oak soils. *Mortierellomycota* was remarkably more present in pine soil.

**Conclusions:**

This analysis of root systems and soil properties confirmed the distinctness of ash stands, which were also more abundant in various microorganisms. It was also found that soils affected by different tree species were characterised by varied fungal and bacterial composition. The ash had particularly beneficial impact on soil microbiota.

## Background

Forests provide numerous benefits for the environment, including climate regulation, water supply, habitats for biodiversity, and erosion control [1–3]. Protecting and restoring forest ecosystems is the key to mitigating climate change and slowing global biodiversity loss [4]. Global change is exposing forest ecosystems to an increased frequency of climate extremes and both the emergence and spread of pests and pathogens [5]. These phenomena affect the stability of forest tree stands. Forest ecosystems can be optimally restored and maintained using climate-based tree species distribution models to predict which tree species will tolerate the climate change [6]. Thus, it is important to understand the influence of different species on soil properties, which, in the future, will facilitate the management of forest ecosystems [7, 8].

Woody vegetation plays an important role in the forest ecosystems and it may affect long-term productivity and sustainability by influencing biochemical processes in the soil environment, such as the microbial activity and diversity, carbon sequestration and nutrient turnover rates [9–11]. Soil properties, such as soil organic carbon, nitrogen and nutrient contents and microbes, not only affect forest growth, but they also modulate ecological soil functionality and biochemical cycles [12, 13]. Changes in aboveground communities can impact the biodiversity of soil microorganisms which, in turn, can be important for regulating the balance between the decomposition and stabilisation of soil organic matter [14]. Soil microorganisms are an important component of biodiversity as they are involved in several ecological processes, with the species composition of the stand being the main factor affecting biodiversity [15]. According to Qiao et al. [16], biodiversity contributes to the stabilisation of forest ecosystems from local to larger spatial scales. Tree species impact soil microbial communities through their litter, roots, mycorrhizal fungi and exudates [17]. Bacteria and fungi living in the soil react differently to the changes in soil characteristics caused by the alterations in the species composition of the stand. According to Guo et al. [18], bacteria are more sensitive to the soil carbon:nitrogen ratio than fungi. The species or compositions of plants in the forest can also play a significant role in promoting the abundance of microbes by shaping their access to nutrients and modifying local environmental conditions [19].

Root systems are vital in shaping soil properties, including microbiological activity [20]. The roots shape the physical, chemical and biological properties of soils through the biomass supplied by dead roots, and their exudates [8, 21]. Root exudates include non-volatile rhizodeposits and soluble organic compounds, such as sugars, amino acids and organic acids [22]. Low-molecular-weight (LMW) root exudates and mucilages can both be used by microbes as a carbon source [23]. Root exudates are considered to be a key determinant of rhizosphere microbial community structure [24, 25].

Due to the unpredictable future threats and the need to improve the stability of forest ecosystems, an alteration in the species composition of stands should be considered. However, the proper reconstruction of forests requires knowledge about the role of individual tree species in shaping the biodiversity of forest ecosystems. So far, several studies have been devoted to the impact of trees on the forest ecosystem and soil environment in particular through the addition of aboveground biomass. There is, however, insufficient knowledge on the impact of the characteristics of root systems on the properties of forest soils and their biodiversity. Our research was focused on bridging this gap.

We examined in detail the root systems of six tree species found in forest stands in the temperate climatic zone. The main objective of our research was to determine the role of root systems in shaping the composition and diversity of soil microorganisms in connection with the basic soil properties. We tested the following research hypotheses: 1) physicochemical properties and enzymatic activity are strongly correlated with the morphological features of the roots of the examined trees, 2) root-exuded carbon positively affects the formation of the physicochemical properties of soils and, consequently, enzymatic activity, 3) ash root systems, together with their exudates, have the most beneficial influence on the properties of the tested soils, 4) the examined tree species, through their root systems and their exudates, have different effects on the soil microorganisms, and 5) coniferous species (pine and larch) have a similar effect on the amount and diversity of fungi and bacteria in the soil.

## Materials and methods

### Study sites

The study was carried out on experimental plots owned by the Department of Ecology and Silviculture at the University of Agriculture in Kraków, Poland. The spaces were located 25 km north of Kraków, in southern Poland (50º11.46.35N, 20º3.54.28E). The study covered the area containing six species of trees–Scots pine (*Pinus sylvestris* L.), European larch (*Larix deciduas* Mill.), English oak (*Quercus robur* L.), English ash (*Fraxinus excelsior* L.), European beech (*Fagus sylvatica* L.) and European hornbeam (*Carpinus betulus* L.). The stands contained single species without admixtures of other species, and were of similar age (70–80 years) and density. Each of the six plot variants (0.1 ha study plots) was tested in five repetitions. In total, 30 study plots were designated for the investigation (six tree species × five repetitions = 30 study plots). On each study plot, five points were designated for the detailed analysis of the root systems and soil properties. Soil and root samples were collected for the analysis from all points. The whole area was characterised by the presence of Luvisols developed from homogeneous loess. The soil samples were collected after the organic horizon was removed from the A horizons, which were 15-cm-thick humus–mineral horizons. Soil samples for laboratory analyses were collected 100 cm from the trunk of trees of the studied species, within the range of their root systems. The collection and analysis was carried out in 2022.

### Chemical analysis

In the collected soil samples, pH in H_2_O and KCl was determined using the potentiometric method. The carbon (C) and nitrogen (N) contents were measured with an elemental analyser (LECO CNS TrueMac Analyzer, Leco, St. Joseph, MI, USA). The P content was measured using a ICP-OES ThermoiCAP 6500 DUO, Thermo Fisher Scientific, Cambridge, U.K.) after mineralisation of the mixture with concentrated nitric and perchloric acids at ratio of 3:1. The cation concentrations (Ca^2+^, Mg^2+^, K^+^, and Na^+^) were extracted with ammonium acetate and determined through the inductively-coupled plasma analysis (ICP-OES Thermo iCAP 6500 DUO, Thermo Fisher Scientific, Cambridge, UK).

### Analysis of enzymatic activity

Enzymatic activity was determined in the soil samples with natural moisture. These samples were sieved through a 2-mm mesh and stored at 4ºC. The activity of six extracellular enzymes––β-glucosidase (BG), β-D-cellobiosidase (CB), β-xylosidase (XYL), N-acetyl-β-D-glucosaminidase (NAG), phosphatase (PH) and arylsulphatase (SP)––was determined using the fluorescence method [26–28].

### Collection of root exudates and analysis of root morphology

The exudates were collected using a culture-based cuvette system [29]. The exudates were collected twice, in June and September 2022. Root exudates were collected from one branched fine root segments of similar length and branching. Each root system was carefully removed, using deionised water and fine forceps, in order to maintain the integrity of the root system. The root systems were placed in sterile glass syringes containing sterile glass beads and moistened with a carbon-free nutrient solution (0.5 mM ammonium nitrate/NH_4_NO_3_, 0.1 mM potassium dihydrogen phosphate/KH_2_PO_4_, 0.2 mM potassium sulphate/K_2_SO_4_, 0.15 mM magnesium sulphate/MgSO_4_ and 0.3 mM calcium chloride/CaCl_2_). After 24 h of stabilisation in the syringe, the roots were flushed three times with fresh carbon-free solution to remove the organic carbon exuded during the stabilisation period. The exudate-containing samples were then collected in 50-mL glass vials that were sealed with silicon caps and stored at 4ºC until we were ready to determine the total organic carbon (TOC).

Trap solutions containing the exudates were collected from each cuvette and filtered through sterile syringe filters. The trap solutions were then analysed using a Shimadzu TOC analyser (Shimadzu, Japan). On each study plot, soil samples with a known volume of 15 × 15 × 15 cm were collected in three replications in order to determine the root biomass. The coarse roots (diameter > 2 mm) were separated from the fine roots (diameter < 2 mm) in these samples. The extracted root system fragments were scanned at 400 dpi resolution and then analysed using a WinRhizo Pro 2003b image analysis system (Regent Instruments Inc., Ville de Québec, QC, Canada) in order to determine their diameter, length and root area. The air-dried roots were further desiccated at 70ºC for 24 h to a constant weight and then weighed. The root tissue density (RTD) (kg m^−3^), specific root area (SRA) (m^2^ kg^−1^) and specific root length (SRL) (m g^−1^) were calculated according to Ostonen et al. [30]. The annual fine root biomass increase was determined using the core method [31]. We sought to determine the root production between April and October 2022.

### Preparation of the soil fungal and bacterial DNA library

The DNA was isolated from the soil picked from the organic (O) horizon (*n* = 3) and from one additional sample from the humus mineral soil (A) horizon. The DNA was isolated from 1 g of soil in accordance with the protocol of the Genomic Mini AX Bacteria + (A&A Biotechnology, Poland). Mechanical lysis was carried out using zirconia balls in FastPrep-24 homogeniser. Lyticase (A&A Biotechnology, Poland) was also used in the enzymatic lysis. Fungal DNA libraries were prepared for the ITS1 rDNA region amplified using ITS1F [32] and ITS2 primers according to the Illumina 16S Metagenomic Library preparation protocol. Bacterial DNA libraries were prepared for the V3–V4 16S rDNA region amplified using 341F and 785R primers [33]. A polymerase chain reaction (PCR) was carried out in a reaction mixture containing 15 ng of genomic DNA using a Q5 Hot Start High-Fidelity 2X Master Mix (New England Biolabs, USA). An indexing PCR was prepared using the Nextera XT index kit (Illumina). After indexing, the samples were purified using AMPure XP beads, verified in a bioanalyser (Agilent Technologies, US) and with qPCR. The DNA libraries were sequenced on an Illumina MiSeq platform (2 × 300 bp paired end) by Genomed (Poland). The sequencing depth was 50 000 reads per sample.

Next-generation sequencing (NGS) data for the fungi were processed using QIIME software [34]. The samples were demultiplexed, and fastq files were generated using MiSeq Reporter v.2.6 software (Illumina). Adapter and low-quality (below Q20) sequences were removed using the cutadapt tool [35]. Paired sequences were joined using the seqprep algorithm. The usearch61 tool was used for chimera removal [36]. The fungal reads were clustered using the uclust algorithm [36] and checked against ITS sequences using the UNITE v.8.2 database (Unite Community 2018) and the BLAST algorithm [37]. Operational taxonomic units (OTUs) were filtered for very low abundance, and only OTUs with a relative abundance of at least 0.01% were used in further analysis. Data on the occurrence of fungal species were used to calculate the relative abundance of fungal types and to generate an abundance heatmap (including Euclidean cluster analysis) using log10 + 1 transformed abundance data for fungal species that totalled more than 500 readings for at least five plots with a given tree species in the Illumina metabarcode. R packages (https://cran.r-project.org) were applied to analyse the microbial diversity and visualise the results. Heatmaps were drawn using the R superheat package.

The NGS bacteria data were processed using QIIME [34]. The samples were demultiplexed, and fastq files were generated using MiSeq Reporter v.2.6. Adapter and low-quality sequences were removed using cutadapt [35]. The paired sequences were joined and clustered using the DADA2 algorithm [38], which removed the chimera. Clustered reads were checked against 16S sequences from the Silva 138 database [39]. Bacterial amplicon sequence variants were processed and analysed as described above for the fungal OTUs. The libraries with the analysed sequences of fungi have been deposited with the Gene Bank https://www.ncbi.nlm.nih.gov/ in project number PRJNA951397 and for bacteria on the number PRJNA956107.

### Statistical analysis

Statistical analyses were performed using the statistical software R (R Core Team 2020) and R Studio (RStudio Team 2020). Spearman correlation coefficients for the soil and root characteristics were calculated. Principal component analysis (PCA) was used to evaluate the relationships between the soil properties and root characteristics. The Shapiro–Wilk test was used to assess normality, and Levene’s test was used to check the homogeneity of the variances. The Kruskal–Wallis test was used to assess the differences between the average values of the soil and root properties.

## Results

### Soil and root characteristics

**Fig. 1:**
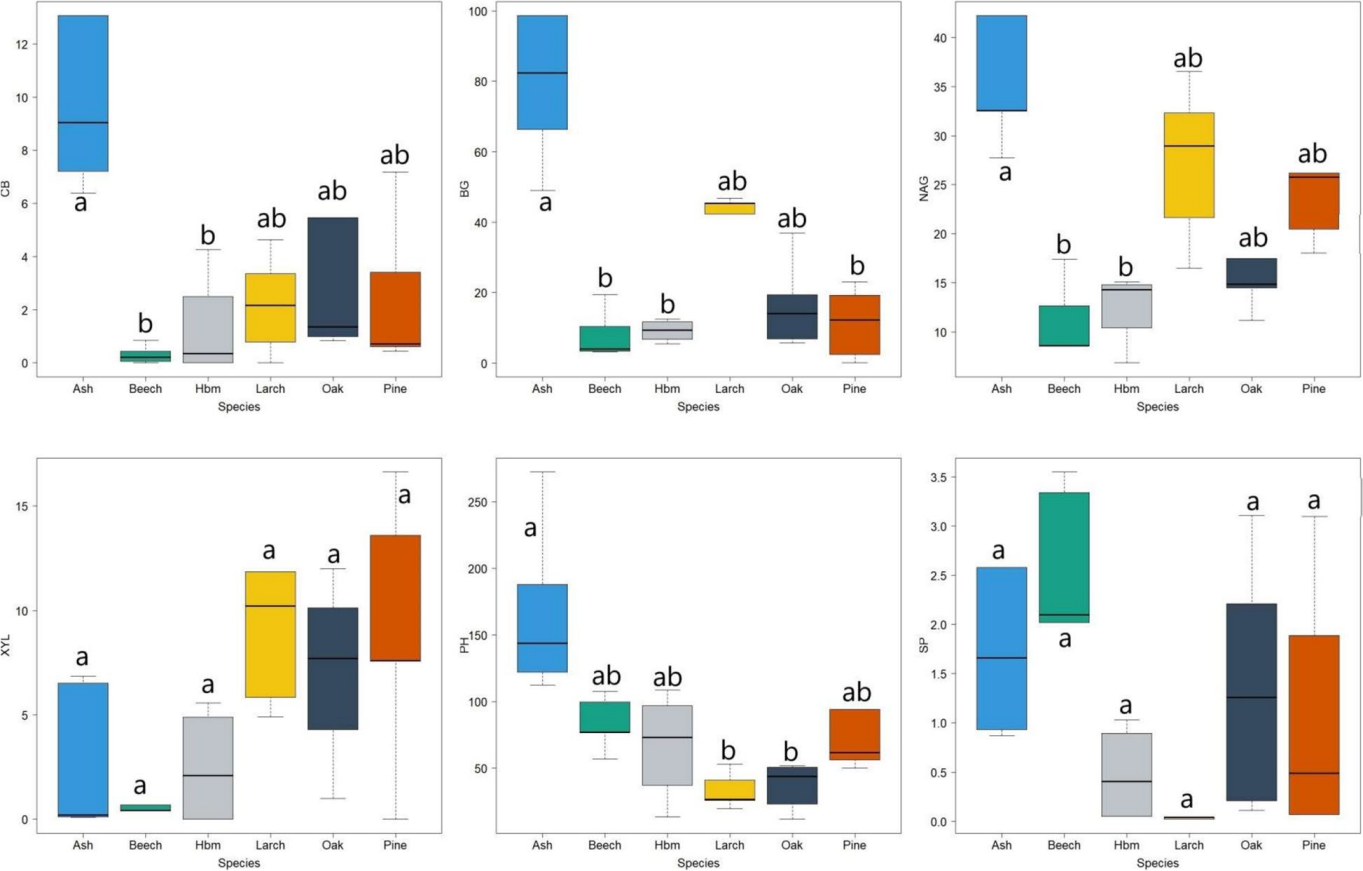
Enzymatic activity (nmol MUB g^−1^ ·C ·h.^−1^) of soils under the influence of various tree species (Hbm – hornbeam; PH—phosphatase, BG—β-glucosidase, NAG—N-acetyl-β-D-glucosaminidase, XYL—β-xylosidase, CB—β-D-cellobiosidase, SP – arylsulphatase; letters (**a**, **b**) mean significant differences between tree species)

The different species investigated in this study had different effects on the physicochemical properties of the studied soils (Table 1). Significantly higher pH values (average pH in H_2_O = 4.86) were found in the soils affected by ash, the lowest in the soils of the larch stand (average pH in H_2_O = 3.71) (Table 1). A significantly higher content of alkaline cations, especially calcium, was recorded in the soils of ash stand. No significant differences in phosphorus and nitrogen content were noted in the soils under the influence of the different species. Significantly higher carbon content (average carbon content = 5.64, 5.19 and 4.75%, respectively) was recorded in the beech, larch and pine stands. The soils of the ash stand were characterised by the lowest (average = 3.63%) carbon content (Table 1). Significantly higher carbon:nitrogen ratios were recorded in the soils with stands of larch (18.6), beech (18.3) and pine (15.4) (Table 1). The soils affected by the different tree species were characterised by having different enzymatic activity (Fig. 1). Significantly higher activity of CB, BG, NAG and PH was recorded in the soils of the ash stands. In terms of XYL and SP activity, there were no significant differences between the studied tree species (Fig. 1).

**Table 1:** Basic properties of soils under the influence of different tree species

	pH H_2_O	pH KCl	Ca (cmol kg^−1^)	K (cmol kg^−1^)	Mg (cmol kg^−1^)	Na (cmol kg^−1^)	P (mg kg^−1^)	N (%)	C (%)	C/N
Ash	4.86 ± 0.51^a^	4.23 ± 0.55^a^	5.65 ± 3.30^a^	0.15 ± 0.09^a^	0.99 ± 0.67^a^	0.05 ± 0.02^a^	388.96 ± 75.71^a^	0.28 ± 0.06^a^	3.63 ± 0.87^b^	12.7 ± 0.4^b^
Beech	4.25 ± 0.12^ab^	3.67 ± 0.02^ab^	1.11 ± 0.43^b^	0.11 ± 0.43^a^	0.22 ± 0.06^a^	0.02 ± 0.01^b^	276.17 ± 16.93^a^	0.31 ± 0.09^a^	5.64 ± 1.48^a^	18.3 ± 0.9^a^
Hornbeam	4.14 ± 0.13^abc^	3.55 ± 0.05^abc^	0.88 ± 0.33^b^	0.89 ± 0.33^a^	0.23 ± 0.05^a^	0.01 ± 0.01^b^	238.46 ± 44.67^a^	0.19 ± 0.03^a^	2.54 ± 0.64^b^	13.7 ± 1.4^b^
Larch	3.71 ± 0.03^bc^	3.14 ± 0.08^bc^	1.21 ± 0.58^b^	1.21 ± 0.58^a^	0.27 ± 0.12^a^	0.04 ± 0.01^a^	367.23 ± 182.71^a^	0.27 ± 0.13^a^	5.19 ± 2.70^a^	18.6 ± 0.8^a^
Oak	3.86 ± 0.13^bc^	3.40 ± 0.09^bc^	1.03 ± 0.44^b^	1.03 ± 0.44^a^	0.33 ± 0.06^a^	0.02 ± 0.01^b^	264.01 ± 65.47^a^	0.26 ± 0.04^a^	3.26 ± 0.87^b^	12.4 ± 1.6^b^
Pine	3.93 ± 0.16^abc^	3.33 ± 0.16^abc^	2.10 ± 0.98^b^	2.10 ± 0.98^a^	0.44 ± 0.13^a^	0.02 ± 0.01^b^	275.51 ± 59.73^a^	0.30 ± 0.07^a^	4.75 ± 1.56^a^	15.4 ± 1.7^a^

Mean ± SD; small letters in the upper index (a, b, c) mean significant differences between different species

**Fig. 2:**
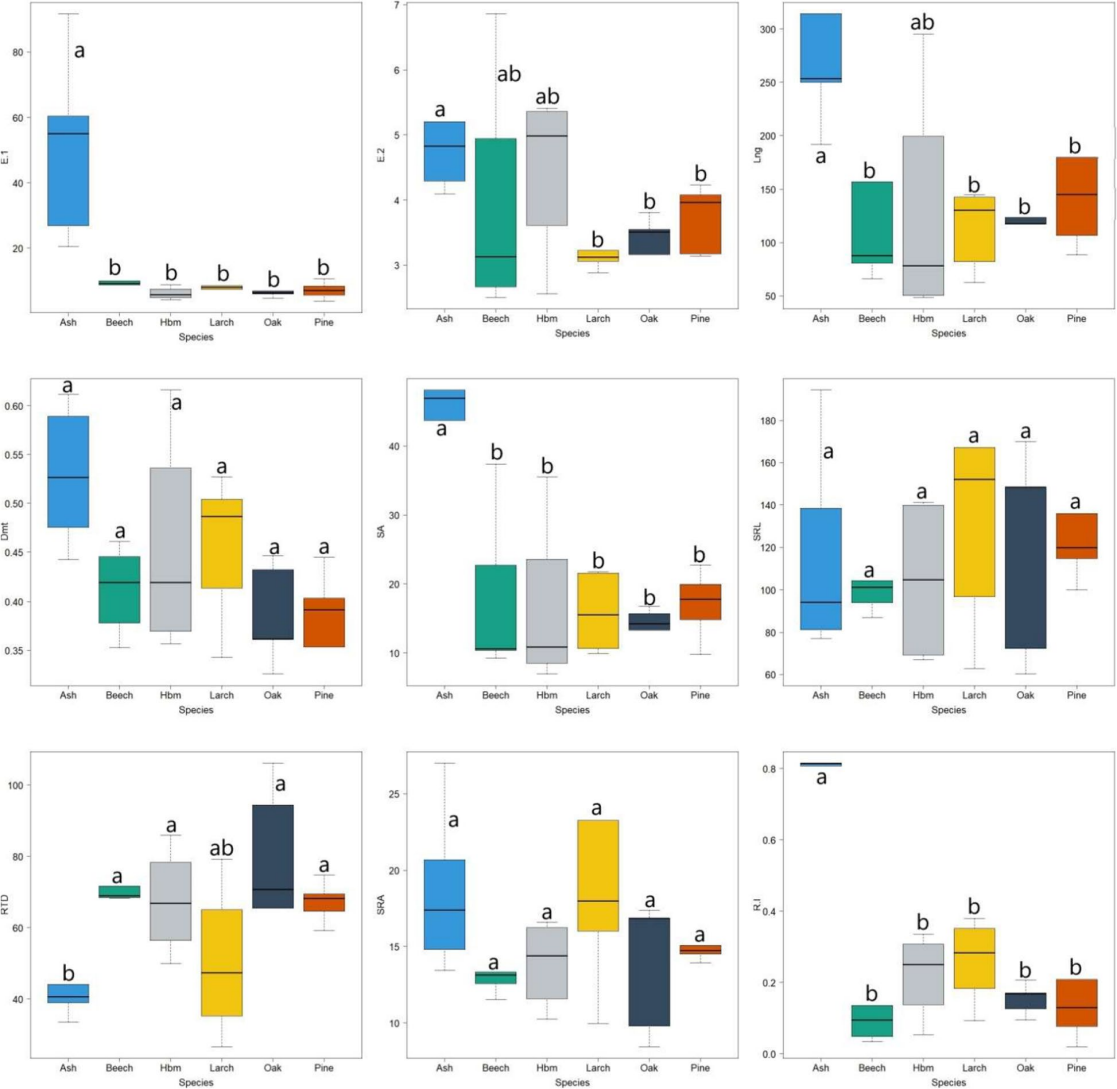
Root characteristics of various tree species covered by the research (Hbm – hornbeam; E.1—root-exuded carbon at the beginning of the growing season (mg C g^−1^ day^−1^), E.2—root-exuded carbon at the end of the growing season (mg C g^−1^ day^−1^), Lng – length roots (cm), Dmt – diameter roots (mm), SA – surface area of roots, SRL—specific root area (m^2^ kg^−1^), RTD—root tissue density (kg m^−3^), SRA—specific root length (m kg.^−1^), R.I – root increase (g); letters (**a**, **b**) mean significant differences between tree species)

**Fig. 3:**
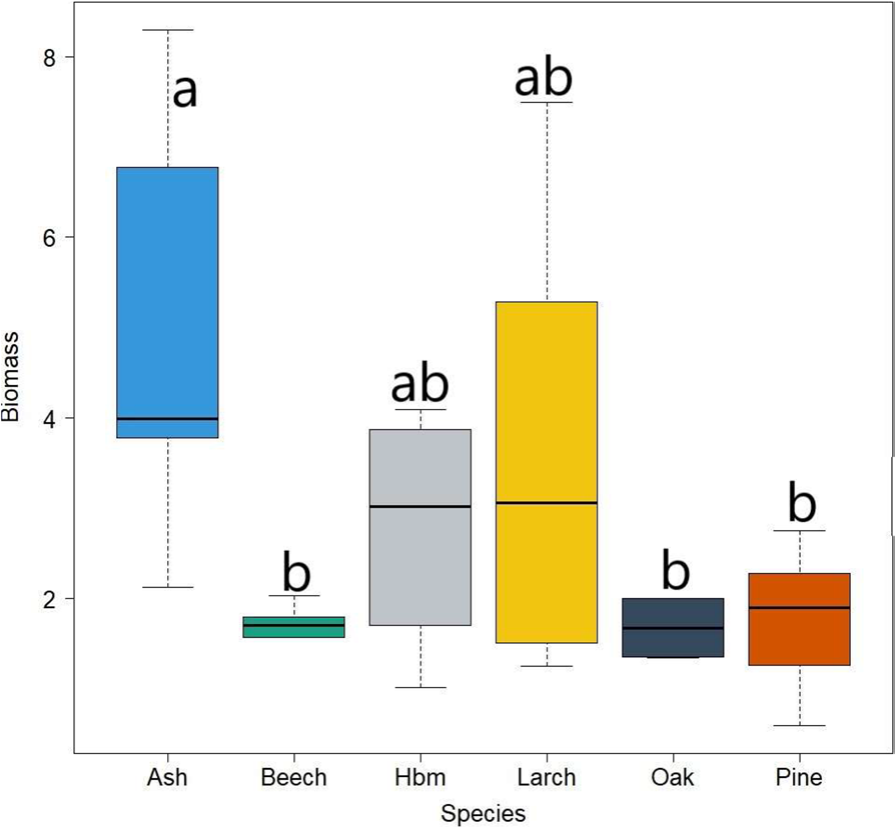
Root biomass (g) of various tree species covered by the research (Hbm – hornbeam; letters (**a**, **b**) mean significant differences between tree species)

Significantly higher amounts of carbon released together with the exudates were in the ash stands (Fig. 2). The root systems of the ash stands differed significantly from the other species in terms of length, diameter and surface area. There were no significant differences between the species in terms of SRL and SRA. Significantly lower RTD was recorded in the ash stands. In addition, a significantly higher root increment was noted in the ash stands (Fig. 2). The ash-root biomass was significantly higher than in the other studied species (beech, oak and pine) (Fig. 3).

**Fig. 4:**
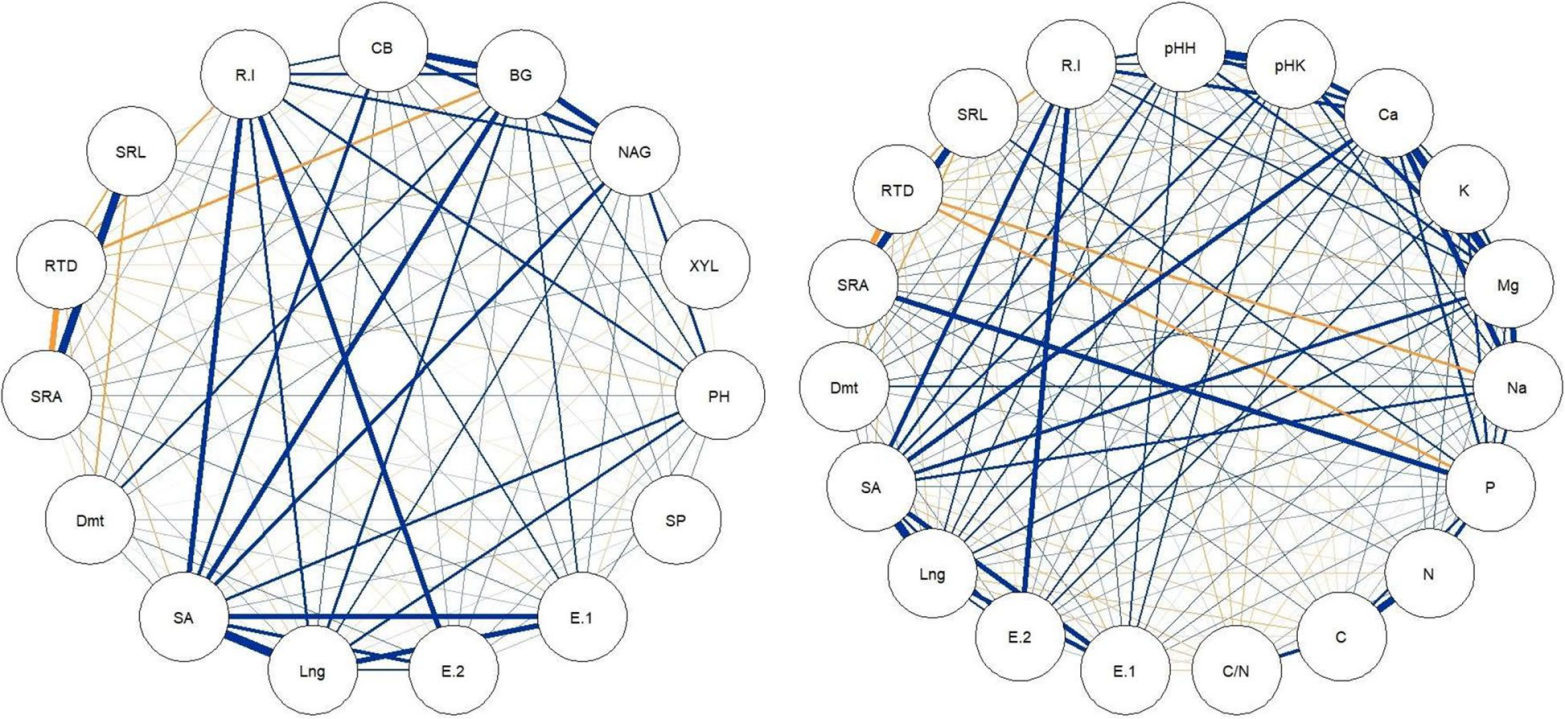
Correlations between root features and soil properties influenced by different tree species (SRL—specific root length, RTD—root tissue density, SRA—specific root area, Dmt – diameter roots, SA – surface area of roots, Lng – length roots, E.1—root-exuded carbon at the beginning of the growing season, E.2—root-exuded carbon at the end of the growing season, R.I – root increase, PH—phosphatase, BG—β-glucosidase, NAG—N-acetyl-β-D-glucosaminidase, XYL—β-xylosidase, CB—β-D-cellobiosidase, SP – arylsulphatase; pHH – pH in H_2_O, pHK – pH in KCl, navy blue—positive correlation, orange—negative correlation)

**Fig. 5:**
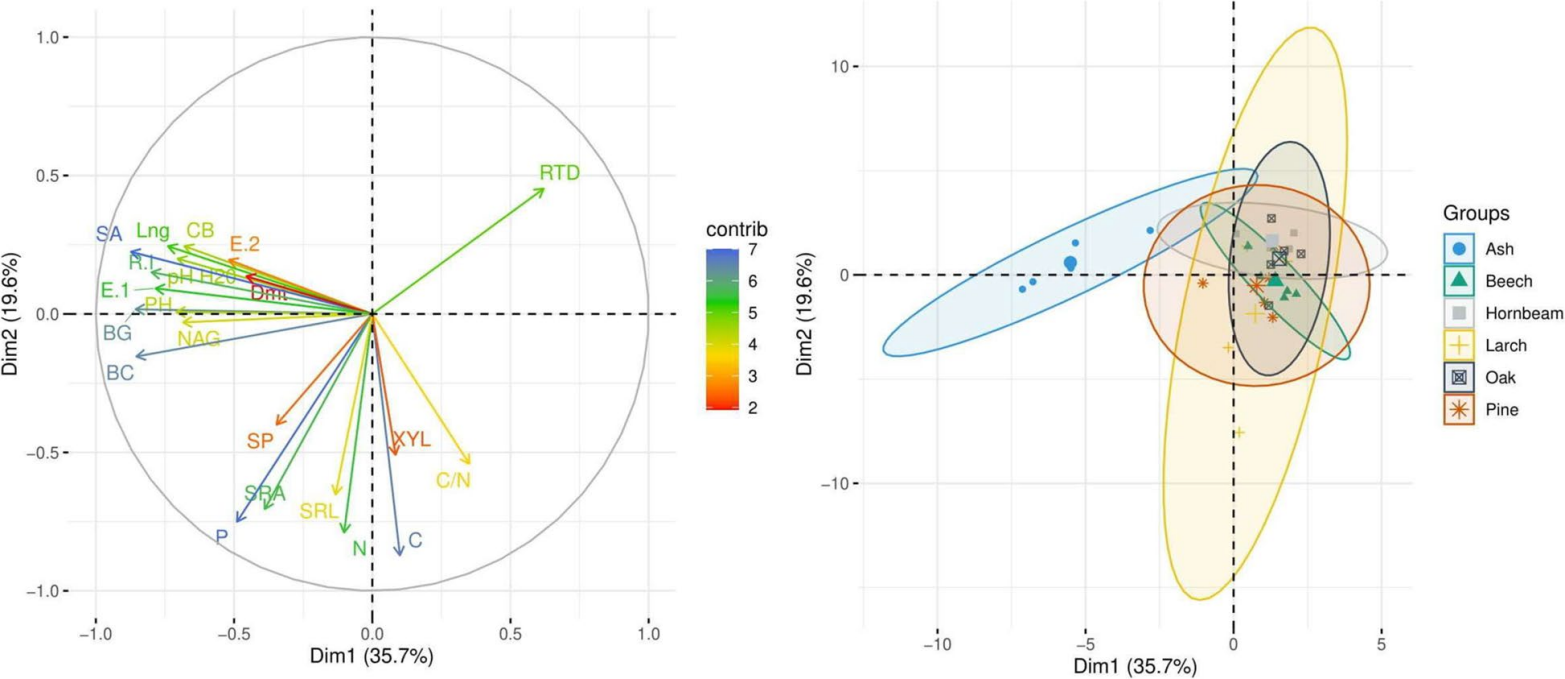
Projection of variables on the plane of the first and second PCA factors (SRL—specific root area, RTD—root tissue density, SRA—specific root length, Dmt – diameter roots, SA – surface area of roots, Lng – length roots, E.1—root-exuded carbon at the beginning of the growing season, E.2—root-exuded carbon at the end of the growing season, R.I – root increase, PH—phosphatase, BG—β-glucosidase, NAG—N-acetyl-β-D-glucosaminidase, XYL—β-xylosidase, CB—β-D-cellobiosidase, SP – arylsulphatase, BC – base cations content)

Statistical analysis confirmed the relationship between the characteristics of the roots and the properties of the tested soils (Fig. 4). Root area and length were strongly positively correlated with the basic cation content. The carbon released with the exudate was positively correlated with pH and the cation content, especially calcium. The SRA and SRL were positively correlated with the phosphorus and, to a lesser extent, the carbon content. A negative correlation was observed between RTD and the content of sodium and phosphorus. Additionally, a strong relationship was observed between the root characteristics and the enzymatic activity in the soils (Fig. 4). The CB, BG, NAG and PH activity strongly positively correlated with surface area and root length, and, to a lesser extent, with diameter. In addition, root growth was strongly positively correlated with the root exudate. A weak positive relationship was noted between the carbon of the exudates and the CB and BG activity. The RTD negatively correlated with BG activity and, to a lesser extent, with NAG activity (Fig. 4). The PCA performed analysis confirmed the relationship between the root characteristics and soil properties (Fig. 5). The PCA analysis explained 45.5% of the variability of the studied features. Factor 1 was especially related to the features of the roots, whereas Factor 2 was related to those features that expressed the quality of soil organic matter. In addition, the PCA confirmed the separateness of root characteristics and soil properties of the ash stands (Fig. 5).

### Fungal and bacterial diversity

**Fig. 6:**
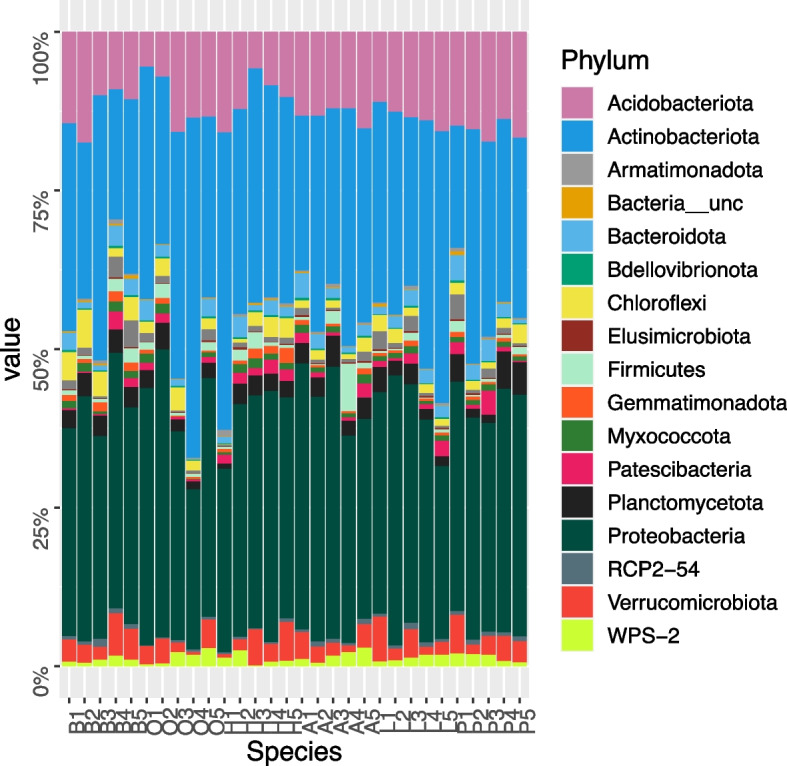
Relative abundance of bacterial phyla based on the Illumina metabarcoding data

The analysis of the share of bacteria phyla in different stand soils revealed that the most numerous phylum was *Proteobacteria*, with the relative share of this phylum being quite stable (amounting to 35.3–37.0%, on average) in the soils inhabited by all the tree species. The second largest group of bacteria was the *Actinobacteriota*, which was more numerous (36.8, 36.4 and 34.4%, respectively) in the soils of oak, hornbeam and larch stands and slightly less numerous (30.8, 29.4, 28.8%, respectively) in the soils of ash, beech and pine stands (Fig. 6). In the case of the *Acidobacteriota*, very similar abundance (10.4–13.3%) was found in the soils of five tree species with the exclusion of pine. In the soils of pine stands, the share of *Acidobacteriota* was slightly higher, amounting to 15.5%. The remaining phylum of bacteria––the *Verrucomicrobiota––*had a share of less than 5%, showing a slightly higher proportion (4.0%) in the soils of the beech stands, whereas it amounted to 2.7–3.6% for the remaining species. The bacteria from the phylum *Planctomycetota* were characterised by a very similar share (2.5–3.6%, on average) in the stand soils. In the case of the phylum *Chloroflexi*, a higher amount (3.9%) was found in the soil of the beech stands, whereas it was 1.4–2.4% for the remaining species. The phylum *Bacteroidota* prevailed (2.5%) in the pine stands, with their share being 1.9–2.1% in the other species. Candidate phylum WPS-2 and the Firmicutes were more numerous (1.7 and 2.3%, respectively) in the soil of the ash stands, while the phylum *Patescibacteria* showed a higher abundance (1.7%) under hornbeam. The phylum *Myxococcota* had a very equal share (0.8–1.1%). The remaining phyla had a share of less than 1% (Fig. 6).

**Fig. 7:**
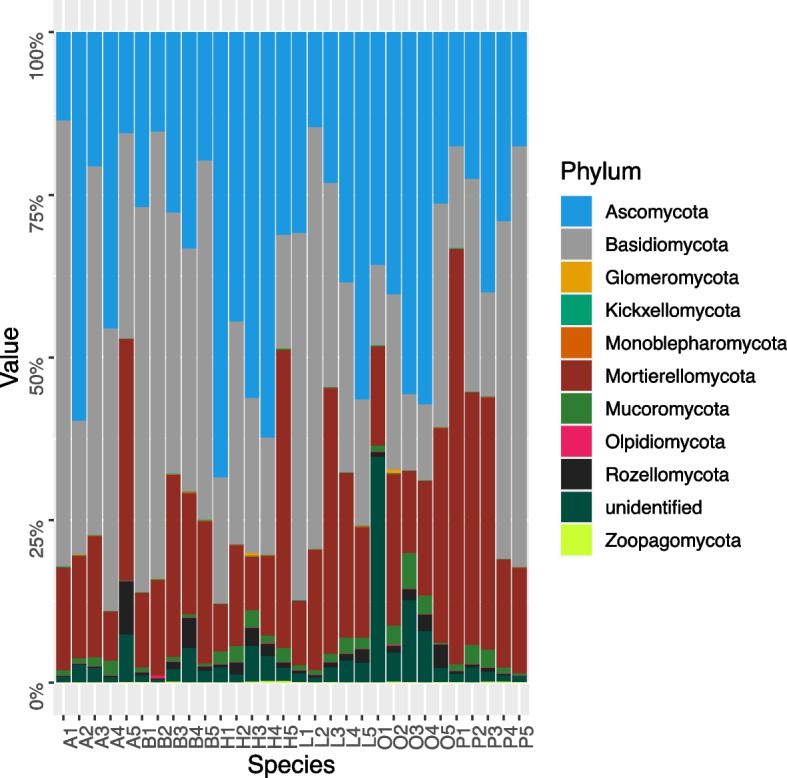
Relative abundance of fungal phyla based on the Illumina metabarcoding data

The analysis of fungal taxonomic units proved that the most numerous phyla in the soils were those of the phyla *Basidiomycota* and *Ascomycota*. The *Basidiomycota* were most numerous (average relative abundance = 52.2%) in the beech soil, followed by the ash (44.2%), larch (40.3%) and pine (36.3%) soils. In the oak and hornbeam stands, the *Basidiomycota* were low in number (19.4 and 22.6%, respectively). The opposite situation was found for the *Ascomycota*, which were most numerous (52.5 and 43.0%, respectively) in the soils under hornbeam and oak, but averaged from 24.5% (beech) to 32.7% (larch) in the other species (Fig. 7). The fungal phylum *Mortierellomycota* was most numerous (35.0%) in the soil of the pine stands, being much lower (19.0% under beech and ash to 22.4% under larch) in the soils of the other species. Relatively high abundances (12.4%, on average) of fungi were identified in the soil of the oak stands whereas, for the other species, the share was much smaller (1.5–3.1%). The remaining fungal phyla were not exceeding 0.1%.

**Fig. 8:**
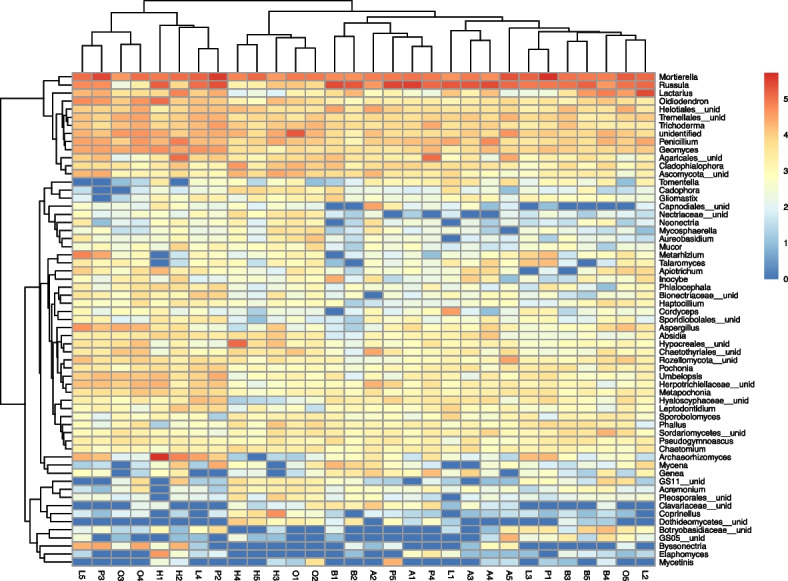
Abundance heatmap based on the Illumina metabarcoding data constructed using log10 + 1 transformed abundance data for fungal species whose total number of reads excided 400

The analysis of fungal taxonomic units using heatmaps showed clear similarities in the structure of the fungal populations between the plots with oak and hornbeam, and the ash and larch stands. The beech stands were similar to the pine stands, the latter being the most diverse in terms of fungal population structure (Fig. 8). Fungi of the genus *Mortierella* were most numerous (34.9%) in the soils of pine stands, and less abundant (17.9–22.4%) in the other cases. The genus *Russula* was most pronounced (35.0 and 33.0%, respectively) under beech and ash, less abundant (23.1% larch, 23.7% pine) in the soils under conifers, and least numerous (3.7 and 4.9%, respectively) under hornbeam and oak. Fungi of the genus *Lactarius* had the highest shares (8.6 and 7.1%, respectively) under larch and beech, whereas its presence was much more insignificant (0.5–2.0%) in the soils under the other species. The remaining types were usually characterised by presence below 5%. The genus *Penicillium* was found to be present in similar amounts (2–4.1%) in the soils of all the studied tree species. Under oak, there was a slightly higher share of fungi from the genera *Oidiodendron*, *Geomyces*, *Aspergillus* and *Trichoderma*, as well as unidentified genera from the orders *Tremellales* and *Helotiales.* Under the hornbeam stands, there was a higher share of fungal genus *Cladophialophara*, as well as unidentified genera from the orders *Agaricales* and *Hypocreales* (Fig. 8)*.*

**Fig. 9:**
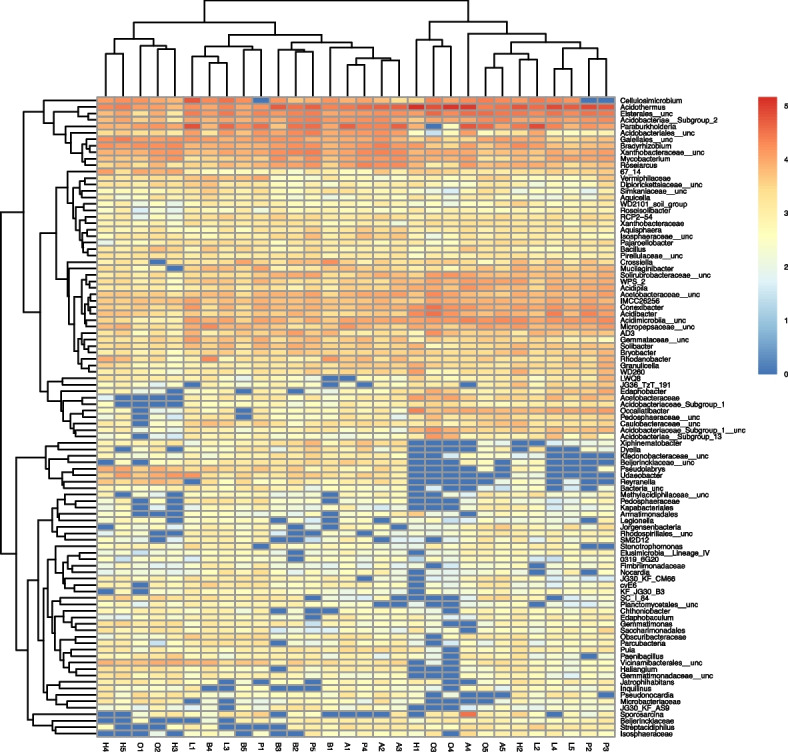
Abundance heatmap based on the Illumina metabarcoding data constructed using log10 + 1 transformed abundance data for bacterial species whose total number of reads excided 400

The analysis of the bacterial population structure showed that the soils contained numerous types of bacteria, each with a relatively low share (Fig. 9). The bacterial genus with the highest relative representation (10.7–11.9%) under all the tree species was *Acidothermus*, except for under beech, where its representation was slightly lower (7.5%). The genus *Paraburkholderia* had a higher abundance (10.3 and 8.2%, respectively) in the soils of larch and ash stands, while its occurrence was lower (2.9–5.9%) for the other species. Also, in the soils under larch, there was a higher percentage (7.6%) of the bacterial genus *Cellulosimicrobium*. Under the beech and oak stands, its share was lower (4.6 and 4.2%, respectively), lower still (2.8 and 2.7%, respectively) under hornbeam and ash, and the lowest (0.9%) under the pine stands. Under the hornbeam stands, there was a slightly higher share (4.3%) of the bacterial genus *Bradyrhizobium*, this share being 1.9–3.0% for the other soils. In the soil under the ash stands, the bacterial genus *Roseiarcus* was most numerous (3.2%), reaching only 0.9–2.6% in the other stands. The remaining types of bacteria were characterised by very low (< 2%) relative abundances. Clusters isolated based on the dominant bacteria (Fig. 9) suggest the presence of three groups of bacterial microbiome surfaces. The microbiomes under the hornbeam stands (H3–H5) shared the greatest similarity to those of the oak stands (O1 and O2). The second large microbiome cluster covered most of the plots containing ash stands (A1–A3), which shared the strongest similarities to all the plots with beech and three plots with pine (P1, P4 and P5). The microbiomes of the larch stands (L2, L4 and L5) were most similar under oak (O3–O5) and pine (P2 and P3) (Fig. 9).

## Discussion

Our results indicate a strong relationship between the morphological features of the roots of the studied tree species and the physicochemical properties of, and enzymatic activity in, the corresponding soils. Root systems are the basic components of the surface horizons of soils and, as such, they have a significant impact on soil physical, chemical and biological properties, mainly via changes in the content of organic matter [40, 41]. Ash root systems, together with their exudates, had the most beneficial effect on the soil properties, differing significantly in terms of root morphology compared with the other species. The ash roots were significantly longer, and had higher surface areas and diameters. Webb et al. [42], in examining how the diversity of root morphology between tree species affected the hydrological properties of soils, found that ash, as a species, could be distinguished by the morphological features of its roots. According to these authors, ash has a great potential for improving the hydrological properties of forest soils because of its roots. In our study, we established a strong positive correlation between the morphological characteristics of the roots and the pH of the soil, as well as the base cation, carbon, nitrogen and phosphorus contents. Ash had the highest annual fine root biomass increase, which undoubtedly had a positive effect on the properties of the soils. According to Shi et al. [43], root biomass increase can change the structure of the soil and its physical properties, especially its hydraulic properties. Numerous studies have shown that longer, faster-growing roots have a greater effect on soil properties such as micropores presence and aggregates formation compared to less developed roots [44]. In the case of ash, we recorded statistically significantly lower RTD compared to the other species, and it is already known that tree-root characteristics are coordinated with, and their suitability is affected by, soil fertility gradients [45], with RTD increasing with decreasing nutrient availability [46] and high RTD being associated with infertile soils [47].

We determined that root-exuded carbon positively affected the physicochemical properties of the soils and, consequently, their enzymatic activity. We also found positive correlations between the root-secreted carbon and the pH of the soils and their enzymatic activity. Root exudates significantly affect the physical and chemical properties of the rhizosphere soil through their complex and diverse composition consisting of three fractions–– diffuses, secretions and excretions [48, 49]. Root exudates have been divided into LMW compounds, which comprise mainly sugars, amino acids and phenol, high-molecular-weight compounds, mainly derived from mucus and extracellular enzymes, and ions [50]. Ash was characterised by the highest amount of root-exudate carbon and the highest enzymatic activity. The development of soil microorganisms is stimulated by providing easily assimilable carbon substrates together with exudates. Our results indicate that exudate composition is related to the tree species, and therefore, through appropriate selection of species composition, it would be possible to influence the soil properties. It is already known from previous studies that root exudates modulate the composition of soil microbial communities by accelerating biochemical reaction, which may improve the decomposition rate processes in soil organic matter [49]. According to Gianfreda [51], all processes and functions occurring in the rhizosphere are predominantly influenced by the activities of plant roots, rhizosphere microorganisms and root–microorganism interactions. Enzymes are recognized as the key players in all activities taking place within this environment. Our results confirm the hypothesis regarding the influence of tree species on the composition of the soil microbiota through their root systems and secretions. In the fungal population structures in the soils hosting the studied tree species, we found differences in the number of fungi specialized in the formation of ectomycorrhizal symbiosis. The taxa that most differentiated the tree species were the fungi *Russula* and *Lactarius*. These genera are known to tend to form ectomycorrhizal associations with a number of tree species in different climatic zones, including the temperate zone [52]. The richness and diversity of soil fungi are strongly related to the tree species composition of the stand [53], whereas the composition and diversity of saprotrophic fungal communities are strongly influenced by external factors, such as pH, the carbon:nitrogen ratio, or soil type and its moisture content [54, 55]. In our plots of different tree species, the soil subtype and its moisture conditions were the same. However, among the tree species, we found differences in the its degree of organic matter decomposition expressed as a C/N ratio, and also some differences in the degree of soil acidity. This might be associated with the impact of the detritus reaching the soil from the individual species, and especially its impact on the root systems. This seems to have been the most important factor influencing the population structure of the soil microorganisms.

The soil fungal and bacterial structures, as well as their functions, may be strongly interconnected [56, 57]. The structure of the fungal population forming mycorrhizal associations could be linked with certain groups of bacteria that are present in root zones, for example, the mycorrhizal zone created by selected *Russula* fungi [58]. The bacterial genera found to be strongly associated with *Russula* mycorrhizae include *Burkholderia*–*Paraburkholderia*, *Mycobacterium*, *Roseiarcus*, *Sorangium*, *Acidobacterium* and *Singulisphaera*. We observed that *Russula* was most prevalent in the beech and ash stands, but in the ash stands*, Burkholderia*–*Paraburkholderia* and *Roseiarcus* were even more abundant. *Paraburkholderia* bacteria, possessing suitable enzymatic abilities, exhibit high phenolic compound degradation activity, thus significantly accelerating the decomposition processes of soil organic matter [59]. *Roseiarcus* bacteria, like ectomycorrhizal fungi, are symbiont that positively impact plant growth and are an indicator of natural, undisturbed microbial environments [60].

At the beginning of our study, we hypothesised that coniferous species such as pine and larch would similarly affect the number and diversity of fungi and bacteria inhabiting the soil. However, this hypothesis was not fully confirmed by our results. The plots with larch, in terms of bacterial structure, had soils more similar to those of ash, while the plots with pine, in terms of bacterial structure, were more similar to the soils of the beech stands. Regarding fungal structure, the plots with pine were quite diverse and did not form a distinct cluster. The plots with pine showed greater similarity in terms of fungal organisms to the larch plots, while forming smaller clusters with the plots of beech, oak and ash stands. The distinctiveness of the soil bacterial community in the larch stands was reflected in the relatively high share of bacteria from the genera *Burkholderia–Paraburkholderia* and *Cellulosimicrobium*, which were less prevalent in the soils of the pine stands. *Cellulosimicrobium* is known for its strong cellulolytic properties [61, 62]. In our study, the soils of the larch stands exhibited a tendency towards higher cellulolytic enzyme activity and selected root parameters (i.e., SRA, RI) compared to the soils of the pine stands. At the same time, the fungal populations in the pine stands were characterised by a large share of the genus *Mortierella*, occurring in lower abundances in the larch stands. *Mortierella* is considered a saprotrophic microorganism, also found in plant root zones, possessing various enzymatic abilities, including the decomposition of polysaccharides (chitin, hemicellulose), enhancing phosphate-ion absorption, and synthesizing phytohormones, all beneficial to plant growth [63]. It should be noted that the conditions of the studied pine stands (on luvisols formed from loess) are considered unnatural (they were artificially introduced by humans), suggesting that differences in the fungal and bacterial structure in this environment might be more reflective of adaptation to new, somewhat unfavourable habitat conditions for the pine stands. The studied pine stands, characterised by good health and no disease symptoms, might confirm their positive adaptation due to a specific set of symbiotic soil microorganisms.

Our findings indicate a positive impact of ash stands on shaping soil properties and enhancing their biodiversity. We observed the beneficial effect of root systems and their exudates on the composition and diversity of microorganisms, as well as on the activity of their enzymes. Currently, in Europe, there is an issue with dying ash stands. The common ash is found in almost all of Europe, and in Poland, it grows throughout the country, except in upper mountain forests [64]. The presence of the fungus *Chalara fraxinea*, the causative agent of ash dieback, has been detected in tissues of dying ash trees [65, 66]. The loss of ash stands could lead to a deterioration of soil properties and, consequently, a decline in the stability of forest ecosystems.

## Conclusions

Our study has confirmed the importance of tree species in shaping soil properties through their root systems. We identified a strong relationship between the morphological features of the roots and the basic physicochemical properties of the soils and their enzymatic activity. Root-exudate carbon was found to be positively correlated with pH, calcium content and the activity of enzymes involved in the carbon and nitrogen cycles. Analysis of the morphological features of the roots and their exudates in connection with soil properties, confirmed the distinctive influence of ash tree stands. We observed differences in the composition of bacterial and fungal associations in relation to coniferous species such as pine and larch. The ash stands were also distinguished by their particular differences in microorganism diversity compared to the other species. The research examining bacteria in the soil of various tree species found that *Proteobacteria* and *Actinobacteriota* were the most prevalent phyla. In the analysis of fungi, *Basidiomycota* and *Ascomycota* emerged as the dominant phyla. The soils under hornbeam and oak trees were particularly rich in *Ascomycota*, while soil of pine trees showed a significant presence of the *Mortierellomycota* phylum. Our findings suggest that the formation of single-species coniferous stands should be avoided, as this leads to a deterioration of soil properties, a reduction in microorganisms diversity, and consequently, a decrease in the stability of the stand. To improve the soil properties and biodiversity, deciduous species such as ash, hornbeam and oak should be introduced into tree stands.

## Data Availability

The data that support the findings of this study are available from the corresponding author on reasonable request. NGS sequence data of bacteria and fungi isolated from soil samples were deposited in the Gene Bank https://www.ncbi.nlm.nih.gov/ in project number PRJNA956107 and PRJNA951397.
